# Comparative evaluation of Oxford Nanopore Technologies’ adaptive sampling and the Twist long-read PGx panel for pharmacogenomic profiling

**DOI:** 10.3389/fphar.2025.1653999

**Published:** 2025-09-09

**Authors:** Koen Deserranno, Laurentijn Tilleman, Dieter Deforce, Filip Van Nieuwerburgh

**Affiliations:** Laboratory of Pharmaceutical Biotechnology, Faculty of Pharmaceutical Sciences, Ghent University, Ghent, Belgium

**Keywords:** pharmacogenomics, long-read sequencing, adaptive sampling, CYP2D6, Twist alliance PGx panel

## Abstract

Clinical pharmacogenomics (PGx) testing strategies are mainly based on targeted PCR, microarrays, or short-read sequencing. These methods perform well for detecting known single-nucleotide variants (SNVs), small insertions/deletions (indels), and certain copy number variants (CNVs), but they fall short in resolving complex structural variants (SVs), particularly in complex pharmacogenes such as *CYP2D6*. Therefore, we previously developed a targeted PGx test based on long-read Oxford Nanopore Technologies (ONT) sequencing. Harnessing adaptive sampling (AS) for *in silico* enrichment of a panel of PGx genes, we illustrated superior performance in star-allele calling compared to the Genetic Testing Reference Materials Program (GeT-RM) truth set. However, accurate diplotyping of *CYP2D6* remained challenging. In this work, we adopted the latest basecalling, variant calling, phasing, and star-allele calling tools on our pre-existing data from the HG001, HG01190, NA19785, HG002, and HG005 reference samples. Additionally, we benchmarked the results to public data obtained using the long-read compatible Twist Alliance PGx panel. The re-analyzed ONT-AS data demonstrated correct *CYP2D6* star-alleles compared to the GeT-RM truth set. Upon benchmarking to the Twist Alliance PGx panel, perfect star-allele matching was obtained between our panel and the Twist PGx panel for all included Clinical Pharmacogenomics Implementation Consortium (CPIC) Level A genes. However, our ONT-AS panel demonstrated superior variant phasing, resulting in three times more variants per phasing block. These findings confirm the robustness of ONT-AS for targeted long-read PGx applications and highlight its potential to support more accurate pharmacogenomic testing, particularly for structurally complex genes like *CYP2D6*.

## 1 Introduction

Pharmacogenomics (PGx) studies the impact of pre-existing DNA variation on the function of medicines. Missense or loss-of-function mutations in protein-coding genes involved in absorption, distribution, metabolization, and excretion (ADME) processes can have profound effects on the drug’s pharmacokinetic and pharmacodynamic parameters. Alternatively, mutations can impact the PGx gene’s expression levels (EMA, 2018). The results of the Pre-emptive Pharmacogenomic Testing for Preventing Adverse Drug Reactions (PREPARE) trial, an international implementation study that investigated the feasibility, acceptability, and effectiveness of panel-based PGx testing, found that 93.5% of all patients carried at least one actionable variant within a 12-gene PGx panel (Swen et al., 2023). Adopting the patient’s genetic information before initiating drug therapy would fully deliver the promise of personalized medicine to achieve better therapeutic outcomes and fewer adverse drug reactions.

PGx is slowly getting incorporated within population healthcare systems. The Estonian BioBank, encompassing both omics-data and health-related information, established PGx microarray genotyping for all of its participants. Based on phenotype translations, recommendations for drug therapy based on nine pharmacogenes for 211,257 individuals are available and will be adopted within the Estonian Health Insurance Fund (Milani et al., 2025). In the Netherlands, the P4Care study, that aimed to study whether DNA-passports result in better therapeutic outcomes for depression and anxiety medication, was recently funded and will include over 2,000 patients (ZonMw, 2025). Additionally, also within clinical guidelines, the recommendations of the Clinical Pharmacogenetics Implementation Consortium (CPIC) and Dutch Pharmacogenomics Working Group (DPWG) are getting adopted, as illustrated by the recommendation for *CYP2C19* genotyping to guide clopidogrel usage by the National Institute for Health and Care Excellence (NICE) and the American Heart Association (Pereira et al., 2024; NICE, 2024).

Most of PGx genotyping is still performed using PCR-based techniques, microarrays, or panel-based short-read sequencing (SRS) (Bourgeois et al., 2024; Bourgeois et al., 2025; Knezevic et al., 2025; Tafazoli et al., 2021; Tayeh et al., 2022). Although these strategies offer high throughput at low cost and provide genotyping of single-nucleotide variants (SNVs), insertions/deletions (indels), and a set of copy number variants (CNVs), they are less suited to elucidate complex genes and to provide haplotype phasing information. Additionally, current PGx assays mostly do not query rare variants or are not able to resolve complex PGx genes that harbor complex structural variants (SVs), high sequence homology, or repeat regions. The *CYP2D6* gene, involved in the metabolization of 25% of the commonly used drugs, is highly polymorphic and notoriously difficult to genotype, due to the presence of two neighboring pseudogenes *CYP2D7* and *CYP2D8* with high sequence homology and complex gene hybrids (Turner et al., 2023). Combined with the observation that only a part of the inherited variability in drug response, even within predicted identical metabolizer phenotypes, can be explained via currently known genetic polymorphisms, it is clear that there is still room for improvement in current genotyping strategies (Zhou et al., 2022; Lauschke et al., 2024; Tremmel et al., 2025).

We recently developed a long-read sequencing (LRS) test that enables the creation of personalized PGx passports, both for pre-emptive and reactive applications (Deserranno et al., 2023). This LRS strategy adopted the unique feature of Oxford Nanopore Technologies (ONT) to dynamically enrich for a prespecified list of target genes without additional steps during library preparation. Using adaptive sampling (AS), we enriched for 1,036 PGx genes extracted from the Pharmacogenomics Knowledge Base (PharmGKB) (Whirl‐Carrillo et al., 2021). We demonstrated improved star-allele calling compared to the Genetic Testing Reference Materials Program (GeT-RM) truth set, findings that were recently corroborated by a group from Singapore (Gan et al., 2025). However, our *CYP2D6* star-allele calls still proved discordant compared to the Get-RM truth set.

The unique advantage of ONT sequencing is that raw squiggle data can be rebasecalled. Our original data were obtained during 2023. Meanwhile, ONT adopted a new raw file format and a novel basecaller yielding increased base call accuracies. Simultaneously, improvements in bioinformatic approaches to variant calling and star-allele calling software were developed. We hypothesized that the combined improvements of rebasecalling and the updated analysis tools might improve the accuracy of *CYP2D6* typing. Moreover, Twist Bioscience recently commercialized a PGx-specific hybridization capture panel compatible with LRS, for which improved *CYP2D6* and *NAT2* genotyping was demonstrated compared to SRS (Barthélémy et al., 2023). Therefore, we additionally performed a direct comparison of our re-analyzed ONT AS PGx data against public data from the Twist Alliance PGx panel.

## 2 Methods

### 2.1 Rebasecalling the ONT data

We re-used the raw sequencing data previously obtained from our AS PromethION sequencing runs (Deserranno et al., 2023). No additional sequencing was performed. In brief, we previously performed targeted ONT sequencing by enriching for 1,036 PGx genes in five Genome in a Bottle (GIAB) reference samples. Rather than physically designing a hybridization capture panel, we used ONT’s AS to selectively enrich library fragments corresponding to the genes of interest during sequencing. On a first PromethION R10.4.1 flow cell, the NA24385 (HG002) and NA24631 (HG005) samples were multiplexed. On a second PromethION flow cell, the NA12878 (HG001), HG01190, and NA19785 were sequenced. The raw .fast5 data were first converted to .pod5 using pod5 convert fast5 (Githup, 2024). Rebasecalling and demultiplexing were performed using Dorado (v0.9.0) (Oxford Nanopore Technologies, 2025). For the first flow cell with HG002 and HG005, the dna_r10.4.1_e8.2_400bps_sup@v4.1.0 model was used. As MinKNOW was upgraded to perform sequencing at 5 kHz instead of 4 kHz in between the sequencing of both flow cells, the second flow cell was rebasecalled using the dna_r10.4.1_e8.2_400bps_sup@v5.0.0 model.

### 2.2 Processing, variant calling, and phasing of the rebasecalled ONT data

From the rebasecalled .fastq files, we selected the reads corresponding to those read_ids enriched by AS. In particular, we filtered the adaptive_sampling.csv file obtained from MinKNOW by only retaining the read_ids with the value “unblock” in the decision column, i.e., retaining the read_ids corresponding to off-target reads. Using grep, we selected all read_ids from the rebasecalled .fastq files that did not correspond to this list of off-target reads. Alignment was performed using minimap2 (v2.28) (Li, 2021) with the GRCh38 reference. For variant calling, we used Clair3 (v1.0.10) (Zheng et al., 2022) with the corresponding pretrained model r1041_e82_400bps_sup_v410 (for HG002 and HG005) and r1041_e82_400bps_sup_v500 (for HG001, HG01190, and NA19785), obtained from Rerio (https://github.com/nanoporetech/rerio). Variant phasing and haplotagging were performed using WhatsHap (v2.4) (Patterson et al., 2015), with flags --include-homozygous --indels --distrust-genotypes --ignore-read-groups.

### 2.3 Star-allele calling using StarPhase

StarPhase (v1.4.0) (Holt et al., 2024) was used for star-allele calling. The phased variant calls and alignment .bam obtained from WhatsHap were used as input. Chromosome naming in the .bam files was adjusted to include the “chr” prefix. StarPhase was run using pbstarphase diplotype --database --bam {bam} –vcf {vcf} --reference {GRCh38} --include-set {list_of_interest} --output-calls PGx_passport.json --normalize-d6-only --output-debug {debug_dir}. The list of interest includes *ABC0G2, CACNA1S, CFTR, CYP1A2, CYP2B6, CYP2C18, CYP2C19, CYP2C8, CYP2C9, CYP2D6, CYP3A4, CYP3A5, CYP4F2, DPYD, G6PD, HLA-DRB1, HLA-B, IFNL3, MT-RNR1, NAT2, NUDT15, RYR1, SLCO1B1,TPMT, UGT1A1,* and *VKORC1*.

### 2.4 Comparison to the Twist Alliance PGx and Dark Genes panel

We retrieved the alignment .bam files for the Twist Alliance PGx and Dark Genes panel from PacBio (https://downloads.pacbcloud.com/public/dataset/). For the Dark Genes panel, we downloaded the dataset for HG001 and HG002, sequenced on the PacBio Revio. For the PGx panel, we downloaded the dataset for HG002 and HG01190, sequenced on the PacBio Sequel II platform. Similarly, we retrieved the alignment .bam files for the Twist Alliance PGx panel sequenced on ONT of sample HG01190 (data available upon request to ONT). The corresponding .bed files listing the targeted gene regions were obtained from the Twist website. For consistency between variant callers, the retrieved .bam files were used as input for Clair3 and WhatsHap with the same settings as listed above. For Clair3 variant calling, the dedicated Clair3 models for Sequel II and Revio data were used.

### 2.5 Read-length and phasing success rate

QC-statistics N50s were obtained using NanoComp (De Coster and Rademakers, 2023). For calculation of the phasing success, the phased .vcf files were filtered to only include the 44 common genes between the ONT-AS PGx panel and the Twist PGx panel using bcftools view (v1.20) (Danecek et al., 2021) by providing a .bed file listing each gene’s start and stop positions. Subsequently, we used WhatsHap (v2.4) stats to obtain the phasing metrics, which were subsequently summarized using multiqc (v1.28) (Ewels et al., 2016). Mosdepth (v.0.3.10) was used for the sequencing depth calculations (Pedersen and Quinlan, 2018).

## 3 Results

### 3.1 Dorado rebasecalling and StarPhase deliver accurate *CYP2D6* star-alleles

Holt et al. recently developed StarPhase, a tool specifically suited to perform diplotyping of PGx genes based on HiFi-sequencing data (Holt et al., 2024). StarPhase allows diplotyping for all genes with CPIC Level A annotation, except *IFNL4*, and a growing number of additional genes. In particular, for genes harboring complex structural variation such as the *HLA*-loci and *CYP2D6*, StarPhase generates consensus haplotypes from the alignment .bam file to call the diplotypes, without supplying a separate .vcf file containing the variant calls. Upon benchmarking the performance of StarPhase to the GeT-RM reference set for 12 common genes, the authors of StarPhase reported 73.8% accuracy. They concluded that the incongruent calls were due to the detection of alleles that were not assayed in the GeT-RM benchmark, updated star-allele nomenclature, or phasing errors in the truth set. These are all known limitations of the GeT-RM reference calls (van der Maas et al., 2025).

Therefore, while developed for PacBio HiFi reads, we tested the performance of StarPhase to call the *CYP2D6* star-alleles based on our existing ONT-AS data. We first directly performed StarPhase on the existing alignment .bam files from our previous research (Table 1, “StarPhase not-rebasecalled ONT data”). Although the star-allele provided for NA19785 is correct, the calls for the other samples remained incorrect. We hypothesized that the lower quality of the provided ONT data confused StarPhase, as marked by the detection of two copies of the *68 allele.

**TABLE 1:** Improvements to CYP2D6 star-allele calling for GeT-RM reference samples. *CYP2D6* core star-allele calls for the ONT-AS PGx panel for five GIAB samples, before and after rebasecalling and processing with StarPhase. The values between parentheses specify the sequencing depth.

	NA12878	HG01190	NA19785	HG002	HG005
GeT-RM reference	*3/*4 + *68	*5/*4 + *68	*1/*2+*13	N/A	N/A
Deserranno et al. (2023) *	*4N.ALDY/*10+*82	*4/*4	*2/*13	*2+*2/*4	*36.ALDY/*49
StarPhase not-rebasecalled ONT data	*3/ *68x2 + *4	*5/ *68x2 + *4	*1/*2+ *13	*2/*4	*36 + *10/*49
StarPhase rebasecalled ONT data	*3/*4 + *68 (25 X)	*5/*4 + *68 (23 X)	*1/*2+ *13 (27 X)	*2/*4 (26 X)	*36 + *10/*49 (23 X)

^a^Alleles ending on .ALDY are custom alleles specific to Aldy software used to call these genotypes and are not part of PharmVar.

As the performance of ONT basecalling has improved in the last 2 years, the raw squiggle data were rebasecalled using the latest Dorado algorithm (*Methods*). Crucially, no new sequencing was performed. Rebasecalling shifted the median read quality Phred score significantly, as illustrated for the NA12878 data, resulting in a shift from 15.5 to 20.4 (Supplementary Figure S1). Supplying the newly basecalled alignment .bam files to StarPhase resulted in correct star-alleles of *CYP2D6* in all samples (Table 1, bottom row). These detected *CYP2D6* star-allele calls are identical to the Get-RM diplotypes and were further documented in the literature using orthogonal LR-sequencing workflows such as CRISPR–Cas9 target enrichment (Bettinotti et al., 2018; Gaedigk et al., 2019; Pratt et al., 2016; Pratt et al., 2010; Rubben et al., 2022). Therefore, together with the results of our previous research using Aldy (Hari et al., 2023) for the other PGx genes, the ONT-AS PGx panel results in an accurate complete long-read PGx passport for nearly all CPIC Level A genes. Only for *HLA-A* (currently not a part of the target file for AS, but can easily be added in our .bed file) and *IFNL4* (currently not covered by StarPhase), calls are not yet included.

### 3.2 Benchmarking the ONT-AS PGx panel to the Twist hybridization panels

The Twist Alliance PGx Panel targets 49 genes, of which 44 are shared with our ONT-AS PGx panel. For future work, *HLA-A* can flexibly be included in our AS .bed file. The other four genes (*CTBP2P2, NAGS, APOL1*, and *GBA)* are absent in the clinical annotations from PharmGKB. Some genes are only partially covered in the Twist PGx panel. For *ADD1*, *CACNA1S*, *CFTR*, *DPYD*, *F2*, *F5*, *GRIK4*, *HTR2C*, *POLG*, and *YEATS4*, the Twist PGx capture probes only target specific parts of the gene, which limits the identification of haplotypes defined by SNVs outside these regions and the detection of novel variants. Focusing on *DPYD*, the Twist PGx panel covers 354 of the 434 annotated polymorphisms included in PharmVar 6.2.5 and, for example, does not include the rs773499329 (MAF 0.1% in the South Asian population) and rs762198241 variants (Pratt et al., 2024; Shrestha et al., 2018). Notably, the mitochondrial encoded *MT-RNR1* gene, included within the CPIC Level A annotations in view of aminoglycoside-induced hearing loss, is not ordinarily included in the Twist PGx panel but may be spiked in, as was the case for the PacBio Twist PGx benchmark discussed below (McDermott et al., 2022). Similarly, it is not included in our ONT AS PGx panel, although it can be flexibly added by appending it to the enrichment .bed file for future experiments. Therefore, and because of the possibility of nuclear mitochondrial DNA segments being falsely aligned to the mitochondrial reference sequence without dedicated enrichment, we did not retain this gene in our analysis (Supplementary Note S1). Twist also supplies the Dark Genes panel, which captures fragments of 389 unique genes, of which 46 are shared with the AS panel list. The three panels share five genes (*HLA-B, HLA-DRB1, IFNL3*, *CYP2D6,* and *VKORC1*) (Supplementary Figure S2).

Of the common genes between the Twist Alliance PGx and ONT PGx panels, 25 (26 including *MT-RNR1*) can currently be diplotyped using StarPhase. The genes include all CPIC Level A genes (except for *HLA-A* and *IFNL4* as discussed above) and *CYP1A2, CYP2C8, CYP3A4, HLA-DRB1, HLA-DQA1*, and *NAT2*. We therefore performed a direct comparison between both strategies using the GIAB reference sample HG01190. Additionally, for this sample, both ONT and PacBio data using the Twist PGx panel are available. A perfect star-allele match was observed across the three datasets, with consistent calls compared to the Get-RM truth set (Table 2). For *CYP2B6, CYP2C9,* and *UGT1A1*, the reference calls were improved, as discussed before (Deserranno et al., 2023). One exception seems to be *HLA-DRB1*, for which both strategies do not correspond to the reference call found by others (Thuesen et al., 2022). Of note, StarPhase was not able to call a diplotype for *HLA-DRB1* based on the ONT sequencing data of the Twist PGx panel, probably due to the lower coverage of the first part of the gene (Supplementary Figure S3). According to the literature, due to the high complexity of the HLA locus, high sequencing depth is required (Thuesen et al., 2022). The sequencing depth for each of the genes profiled can be found in Supplementary Table S1. The median sequencing depths along *HLA-DRB1* were 15.79 X and 19.62 X, for ONT-AS PGx and Twist PacBio, respectively, most probably explaining the discording call. Of note, for *HLA-DQA1*, a minor discrepancy regarding a difference in the non-coding region is found between ONT-AS PGX and PacBio Twist on one side and ONT Twist on the other side. As historical methods do not allow eight-digit HLA discrimination, we could not retrieve a comprehensive reference call for this sample; hence, we cannot accurately determine which one is correct.

**TABLE 2:** PGx passport for HG01190, as established by ONT-AS PGx, and the Twist Alliance PGx panel sequenced on PacBio Sequel II and ONT. The results were compared to the those of the Get-RM Truth set, where possible. Dark green: improvement of the truth set; light green: concordance with the truth set; no fill: no truth set call available. Only the major star-alleles were considered. For *CYP1A2 and CYP3A4,* the minor allele suffix A or B is outdated.

Gene name	Get-RM truth set	PGx ID ONT as PGx	PGx ID PB twist	PGx ID ONT twist
*ABCG2*	-	rs2231142 G/G	rs2231142 G/G	rs2231142 G/G
*CACNA1S*	-	Ref/Ref	Ref/Ref	Ref/Ref
*CFTR*	-	Ref/Ref	Ref/Ref	Ref/Ref
*CYP1A2*	*1A/*1A	*1/*1	1/*1	1/*1
*CYP2B6*	*1(*5)/*1(*27)	*1/*5	*1/*5	*1/*5
*CYP2C19*	*1/*2	*1/*2	*1/*2	*1/*2
*CYP2C8*	**1/*3	*1/*3	*1/*3	*1/*3
*CYP2C9*	*2/*61	*1/*61	*1/*61	*1/*61
*CYP2D6*	*5/*4+*68	*5/*68 +*4	*5/*68 +*4	*5/*68 +*4
*CYP3A4*	*1/*1B	*1/*1	*1/*1	*1/*1
*CYP3A5*	*1/*1	*1/*1	*1/*1	*1/*1
*CYP4F2*	*1/*3	*1/*3	*1/*3	*1/*3
*DPYD*	*1/*9	*9A/Ref	*9A/Ref	*9A/Ref
*G6PD*	NEG	B/B	B/B	B/B
*HLA-B* ^a^	*15:20/*18:01:01G	*15:20/*18:01:01:83	*15:20/*18:01:01:83	*15:20/*18:01:01:83
*HLA-DRB1* ^a^	*04:04:01/*08:01:01G	*08:01:01:01/*08:01:01:01	*08:01:01:03/*08:01:01:03	No reads
*HLA-DQA1* ^a^	*03:01:01G/*04:01:01G	*04:01:01:03/*03:01:01:01	*04:01:01:03/*03:01:01:01	*04:01:01:01/*03:01:01:01
*IFNL3*	C/C	rs12979860 C/C	rs12979860 C/C	rs12979860 C/C
*NAT2*	*4/*4	*4/*4	*4/*4	*4/*4
*NUDT15*	*1/*1	*1/*1	*1/*1	*1/*1
*RYR1*	-	Ref/Ref	Ref/Ref	Ref/Ref
*SLCO1B1*	*1/*1	*1/*1	*1/*1	*1/*1
*TPMT*	*1/*1	*1/*1	*1/*1	*1/*1
*UGT1A1*	(*37)/*60	*1/*80+*37	*1/*80+*37	*1/*80+*37
*VKORC1*	*H7/*H7	rs9923231 C/C	rs9923231 C/C	rs9923231 C/C

^a^ *HLA-B, HLA-DRB1,* and *HLA-*DQA1 were consulted from Thuesen et al. (2022). These were colored as concordant if their two-field (4-digit) codes were matching.

Additionally, we repeated the star-allele calling benchmark for HG002 and found near-perfect agreement between both strategies (no Get-RM truth set available), except for the suballele of *CYP2D6*, the non-coding variant in *HLA-DRB1* and one *HLA-DQA1* genotype (Supplementary Table S2). *CYP2D6* suballeles do not convey any additional functional impact compared to the major star-allele. The CYP2D6*4.014 suballele detected in the ONT-AS PGx panel is different from the CYP2D6*4.015 suballele detected using the Twist panel in the presence of rs1473203326 in the *4.014 allele. The presence of this SNV could not be deduced from manual inspection of the ONT-AS PGx reads, pointing to a possible artifact in StarPhase (Supplementary Table S2 and Supplementary Figure S4). The disagreeing HLA allele calls are discussed below.

Furthermore, we assessed the usability of the Twist Dark Genes panel for PGx as it shares five genes with the ONT-AS PGx panel and the Twist PGx Panel (Table 3, Supplementary Table S3). Strikingly, the star-allele call for *CYP2D6* and *IFNL3* for HG001 is wrong using the Twist Dark Genes panel. Upon closer examination, the capture region of *CYP2D6* in this panel is too limited to reliably call the diplotype. The limited capture region is especially problematic for samples harboring complex *CYP2D6* gene configurations, involving non-identical gene duplications and hybrids with neighboring pseudogenes, as is the case for HG001. Comparing the capture region of *CYP2D6* between the Twist Dark Genes and Twist PGx panel, the PGx panel also captures the neighboring regions of *CYP2D6*, which might explain the wrong call with the Dark Genes panel (Supplementary Figure S5). For samples with non-complex *CYP2D6* gene configurations such as HG002, our results indeed confirm that this limited capture region is less problematic (Table 3). Similarly, for *IFNL3*, the capture region included in the Dark Genes panel is too limited to reliably detect all possible star alleles (Supplementary Figure S6).

**TABLE 3:** Head-to-head comparison of the common and StarPhase supported genes between the ONT AS PGx, PacBio Twist Dark Genes, and PacBio Twist PGx panels. For HG002, no Get-RM reference data were available. Light green: concordant reference call; orange: incorrect call compared to reference.

	HG001			HG002			
Gene name	Reference	ONT as PGx	PacBio twist dark genes	Reference	ONT as PGx	PacBio twist dark genes	PacBio twist PGx
*CYP2D6*	*68+*4.001/*3	*68+*4.001/*3	*3/*4.001	-	*2.001/*4.014	*2.001/*4.015	*2.001/*4.015
*HLA-B* ^a^	*56:01/*08:01	*56:01:01:04/*08:01:01:01	*56:01:01:04/*08:01:01:01	38:01:01:01/35:08:01:01	*38:01:01:01/*35:08:01:01	*38:01:01:01/*35:636 N	*38:01:01:01/*35:08:01:01
*HLA-DRB1* ^a^	*01:01/*03:01	*03:01:01:24/*01:01:01:01	01:01:01:01/*01:01:01:04	10:01:01:03/04:02:01	*10:01:01:03/*10:01:01:03	*04:02:01/*10:01:01:01	*10:01:01:04/*10:01:01:04
*IFNL3*	C/T	rs12979860 C/T	rs12979860 C/C	-	rs12979860 C/C	rs12979860 C/C	rs12979860 C/C
*VKORC1*	*H1/*H9	rs9923231 C/T	rs9923231 C/T	-	rs9923231 T/T	rs9923231 T/T	rs9923231 T/T

^a^ HLA-B and HLA-DRB1 diplotypes for HG001 and HG002 were obtained from Dilthey et al. (2016) and Lai et al. (2024), respectively. Boxes were colored as concordant if their two-field (4-digit) codes were matching.

The results for HLA typing across the different panels are more complex (Table 3). *HLA-B* and *HLA-DRB1* reference calls for HG001 and HG002 were obtained from the literature (Lai et al., 2024; Chin et al., 2020). For HG001, the ONT-AS PGx panel demonstrates concordance with the reference, while *HLA-DRB1* is most probably false for the Twist Dark Genes panel. Manual curation (Supplementary Figure S7) indicated that the increased aligned read length in the ONT-AS PGx panel compared to the Dark Genes panel might have contributed to the correct *HLA-DRB1* gene assignment. Despite the full inclusion of *HLA-B* in the Dark Genes panel, the *HLA-B* call for HG002 with this panel is most likely wrong, probably due to the limited sequencing depth (Supplementary Figure S8). *35:636 N differs from *35:08:01:01 only by a C→T substitution in gDNA position 331 of exon 2 (Loginova et al., 2025). For *HLA-DRB1*, strikingly, only the Dark Genes panel seems to be concordant with the literature reference benchmark reported by Lai et al. (2024). Of note, one of the analysis tools used in the construction of their benchmark, i.e., HLA-VBseq, did output the HLA-DRB1*10:01:01:03/*10:01:01:03 diplotype retrieved by StarPhase for the ONT-AS PGx dataset. For *HLA-DRB1*, the Twist Dark Genes panel had a median sequencing depth of 30 X compared to 21 X both for the ONT-AS PGx and Twist PGx panels. Conversely, the Dark Genes panel had the lowest sequencing depth for *HLA-B* (median sequencing depth: 19 X) compared to the ONT-AS PGx panel (44 X) and Twist PGx panel (143 X), therefore most probably explaining the assumed wrong calls. Our results confirm that high coverage is crucial for HLA-typing, more than for other PGx genes, and longer read lengths might help in calling the correct allele.

**FIGURE 1:**
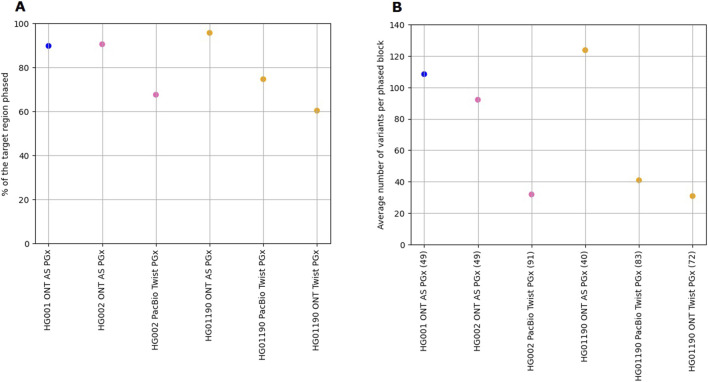
Benchmarking the level of phasing between the ONT-AS PGx panel and the Twist PGx panel across the HG001, HG002, and HG01190 GIAB samples. **(A)** The percentage of the target region that could be phased, calculated as the number of bases phased relative to the total number of bases targeted within the panel. **(B)** The average number of variants per phased block. The value between parentheses next to the sample names in the horizontal axis represents the number of phased blocks.

Finally, we benchmarked the overall read length and variant phasing capabilities between the ONT-AS PGx and the Twist PGx panel data. Due to the nature of the hybridization capture library preparation, the aligned N50 read length for the hybridization panels is significantly smaller (ONT Twist PGx: 4,040 bp; PacBio Twist PGx: 5,370 bp) than the native ONT-AS PGx reads (8,047 bp), as profiled for HG01190. Corroborating the findings of previous research, this also impacted the level of the target region that can be phased. For the 44 common genes, the ONT-AS PGx panel had significantly larger proportions of the target gene loci phased (Figure 1A) than the Twist PGx panel. The percentage of the targeted region phased for the Twist PGx panel was similar to previously reported results (van der Lee et al., 2022). Additionally, the number of individual phasing blocks for the ONT-AS PGx panel was approximately half of the number of blocks in the Twist PGx data, and each phasing block included more variants (Figure 1B). For the ONT-AS PGx panel, the maximum read length that can be obtained theoretically only depends on the length of the input DNA molecules. For this research, no shearing was performed. However, longer DNA fragments tend to cause increased blocking of the nanopores, and DNA shearing to, for example, 10 kb has been proposed to address this. Although shearing might boast yield during ONT AS and allow more samples to be multiplexed on a single-flow cell, it limits the number of unique variants contained within a single read available for phasing.

## 4 Discussion

The reanalysis of the ONT-AS PGx panel and the benchmarking results of the targeted Twist PGx panel illustrate the high potential of long-read PGx for clinical use. Both panels resulted in accurate or improved star-allele calls compared to the Get-RM truth. For the ONT-AS PGx data, reanalyzing the previously generated data with the latest software versions and leveraging StarPhase for star-allele calling now yielded correct *CYP2D6* star-alleles compared to our previous research. Based on our comparison, the Twist Dark Genes panel is not suitable for pharmacogenomics research, due to the very limited inclusion of key gene regions. As discussed by others, another advantage of using LR sequencing is the phasing of variants to the allele-of-origin, which is particularly important for genes such as *SLCO1B1* and *CYP2B6* as they list star-alleles with overlapping variants which, without phasing information, can be wrongly assigned (Desta et al., 2021; Ramsey et al., 2023; van der Lee et al., 2020). Our results illustrate the superiority of the ONT-AS PGx panel in variant phasing and read length.

The Twist PGx panel and the ONT-AS PGx panel both have their unique properties. Considering the number of genes, the Twist PGx panel is limited to 50 genes, some not fully covered, compared to the ONT-AS PGx panel, currently covering 1,036 full-length genes with any level of evidence in the PharmGKB clinical annotation list. However, the genes included in the Twist PGx panel cover all CPIC Level A evidence genes, except for *IFNL4*. The ONT-AS PGx panel offers the additional advantage of flexibly adding genes to the .bed file, considering that, for successful performance of AS, the target panel should be large enough to prevent the nanopores from wearing out quickly. Regarding the throughput, PacBio states that 72 Twist PGx Panel-enriched samples could be sequenced at once on a single Revio SMRT cell, whereas ONT reports that up to 48 samples could be multiplexed on a PromethION flow cell, compared to three samples using AS (Deserranno et al., 2023; Whirl‐Carrillo et al., 2021; Oxord Nanopore Technologies, 2025; PacBio, 2023). However, ONT-AS requires no additional reagents, hands-on time, cost, and can be performed completely PCR-free, while the hybridization procedure for Twist involves a multi-day protocol for library preparation, 16 h overnight incubation, and pre- and post-capture long-range PCR-amplification steps. The use of PCR steps also limits the potential of using 5-methylcytosine and 5-hydroxymethylcytosine detection, which can directly be retrieved from the ONT AS PGx data.

The ONT-AS PGx data have been demonstrated to be future-proof. Rebasecalling of already existing data provided increased accuracy and combined with the latest algorithms for phasing, variant calling, and star-allele calling have now yielded a complete PGx passport. However, for HLA typing, increased sequencing depth or improved analysis pipelines are needed. Currently, the provided star-alleles can be used as input, for example, PharmCAT to provide phenotypes and dosing recommendations. However, for some star-alleles, such as, *CYP2D6*, the impact is unknown. Modeling the enzymatic activity on a continuous scale, rather than the categorical star-allele classification based on long-read sequencing data, has shown promising results for *CYP2D6* (van der Lee et al., 2021).

In conclusion, we demonstrate that our ONT-AS PGx strategy enables accurate diplotyping for common and actionable PGx genes. In particular, for the *CYP2D6* gene, correct star-alleles are now retrieved. We compared the performance of our strategy against the Twist Alliance PGx panel, in combination with PacBio or ONT LRS, and demonstrated concordant star-allele calls for both panels. For HLA-typing, increased sequencing depth and long read lengths contribute to accurate genotyping. Although it is not the primary objective, we also assessed the performance of the Twist Dark Genes panel for PGx profiling and found it to be less suited for PGx purposes. Furthermore, we identified unique advantages for each strategy. Although the hybridization panels claim higher multiplexing of samples, the ONT-AS strategy demonstrates more comprehensive gene profiling in a less labor-intensive workflow and superior variant phasing information. Overall, we highlight the potential of targeted long-read PGx applications and its potential to support more accurate clinical PGx testing.

## Data Availability

Publicly available datasets were analyzed in this study. These data can be found here: The datasets analyzed for this study can be found in Array Express under accession E-MTAB-15248. The datasets analyzed for our previous study can be found on https://www.ncbi.nlm.nih.gov/bioproject/PRJNA1003794.
