# Supplementary material for: Comparative evaluation of Oxford Nanopore Technologies’ adaptive sampling and the Twist long-read PGx panel for pharmacogenomic profiling
Source: Front Pharmacol. 2025 Sep 9;16:1653999. doi: 10.3389/fphar.2025.1653999 (PMC12455207; doi:10.3389/fphar.2025.1653999)
Supplement: Supplementary file 1 [file DataSheet1.pdf]

Supplementary Materials

Contents

1.1 Supplementary Tables 2
1.1.1 Supplementary Table S1 2
1.1.2 Supplementary Table S2 3
1.1.3 Supplementary Table S3 4
1.2 Supplementary Figures 5
1.2.1 Supplementary Figure S1 5
1.2.2 Supplementary Figure S2 6
1.2.3 Supplementary Figure S3 7
1.2.4 Supplementary Figure S4 8
1.2.5 Supplementary Figure S5 9
1.2.6 Supplementary Figure S6 10
1.2.7 Supplementary Figure S7 11
1.2.8 Supplementary Figure S8 12
1.3 Supplementary Notes 13
1.3.1 Supplementary Note S1 13

1.1 Supplementary Tables

1.1.1 Supplementary Table S1

Table S.1 Sequencing depths* for the genes profiled for HG01190 across the different strategies

	HG01190		
	**PGx ID ONT AS PGx**	**PGx ID PB Twist**	**PGx ID ONT Twist**
ABCG2	32.29	91.32	254.62
CACNA1S	29.77	22.51	52.71
CFTR	30.74	1.28	4.51
CYP1A2	33.95	169.38	390.53
CYP2B6	36.85	115.59	277.84
CYP2C19	29.09	46.78	108.99
CYP2C8	30.3	61.42	229.19
CYP2C9	30.77	69.19	220.11
CYP2D6	23.34	100.73	137.78
CYP3A4	37.89	136.47	547.28
CYP3A5	31.38	120.21	467.47
CYP4F2	26.89	76.43	198.61
DPYD	31.8	11.47	39.48
G6PD	16.87	70.17	172.04
HLA-B	41.23	151.59	664.37
HLA-DQA1	32.04	25.99	17.96
HLA-DRB1	15.79	19.62	45.31
IFNL3	31.12	35.76	140.17
MT-RNR1	17.39	4771.19	3583.94
NAT2	31.45	104.13	417.43
NUDT15	31.59	98.17	443.54
RYR1	28.12	121.14	282.36
SLCO1B1	31.84	96.86	353.58
TPMT	28.46	109.3	316.89
UGT1A1	30.3	100.69	351.77
VKORC1	39.59	63.72	117.64

*Sequencing depth is calculated using mosdepth (v0.3.10) using the gene’s start and end coordinates as boundaries.

1.1.2 Supplementary Table S2

Table S.2 Results of StarPhase for HG002 using the AS ONT PGx and Twist PGx panel

Gene Name	PGx ID ONT AS		PGx ID PacBio Twist	
	**Diplotype**	**Depth***	**Diplotype**	**Depth***
ABCG2	rs2231142 reference (G)/ rs2231142 reference (G)	31.89	rs2231142 reference (G)/ rs2231142 reference (G)	79.63
CACNA1S	Reference/Reference	31.74	Reference/Reference	18.11
CFTR	ivacaftor non-responsive CFTR sequence/ivacaftor non-responsive CFTR sequence	35.61	ivacaftor non-responsive CFTR sequence/ivacaftor non-responsive CFTR sequence	1.07
CYP1A2	*30/*30	30.84	*30/*30	133.51
CYP2B6	*2/*5	26.29	*2/*5	90.35
CYP2C19	*1/*1	30.71	*1/*1	41.33
CYP2C8	*1/*1	35.23	*1/*1	60.94
CYP2C9	*1/*1	33.25	*1/*1	63.83
CYP2D6	*2.001/*4.014	26.05	*2.001/*4.015	107.81
CYP3A4	*1/*1	34.98	*1/*1	122.96
CYP3A5	*3/*3	27.52	*3/*3	122.92
CYP4F2	*4/*5	28.03	*4/*5	67.41
DPYD	Reference/Reference	32.99	Reference/Reference	10.68
G6PD	B (reference)/B (reference)	11.91	B (reference)/B (reference)	58.13
HLA-B	*38:01:01:01/*35:08:01:01	43.5	*38:01:01:01/*35:08:01:01	143.15
HLA-DRB1	*10:01:01:03/*10:01:01:03	21.07	*10:01:01:04/*10:01:01:04	21.36
HLA-DQA1	*01:05:01:01/*03:01:01:01	39.08	*01:05:01:01/*01:05:01:01	51.15
IFNL3	rs12979860 reference (C)/ rs12979860 reference (C)	21.48	rs12979860 reference (C)/ rs12979860 reference (C)	30.77
MT-RNR1	Not included in target .bed file	306.69	Reference/Reference	6117.52
NAT2	*4/*6	31.73	*4/*6	100.68
NUDT15	*1/*1	40.04	*1/*1	92.71
RYR1	Reference/Reference	28.64	Reference/Reference	106.41
SLCO1B1	*1/*1	30.92	*1/*1	90.45
TPMT	*1/*1	27.56	*1/*1	85.53
UGT1A1	*1/*1	25.5	*1/*1	108.8
VKORC1	rs9923231 variant (T)/ rs9923231 variant (T)	29.26	rs9923231 variant (T)/ rs9923231 variant (T)	49.22

*Sequencing depth is calculated using mosdepth (v0.3.10) using the gene's start and end coordinates as boundaries.

1.1.3 Supplementary Table S3

Table S.3 Sequencing depths* for the common genes between the Twist PGx, Twist Dark Genes and ONT AS PGx panel.

	HG001		HG002		
**Gene**	**ONT AS PGx**	**PacBio Twist Dark Genes**	**ONT AS PGx**	**PacBio Twist Dark Genes**	**PacBio Twist PGx**
CYP2D6	25.25	73.51	26.05	54.69	107.81
HLA-B	27.97	35.64	43.5	18.82	143.15
HLA-DRB1	18.39	81.6	21.07	30.31	21.36
IFNL3	17.5	14.25	21.48	13.51	30.77
VKORC1	18.72	24.67	29.26	13.16	49.22

*Sequencing depth is calculated using mosdepth (v0.3.10) using the gene's start and end coordinates as boundaries.

1.2 Supplementary Figures

1.2.1 Supplementary Figure S1

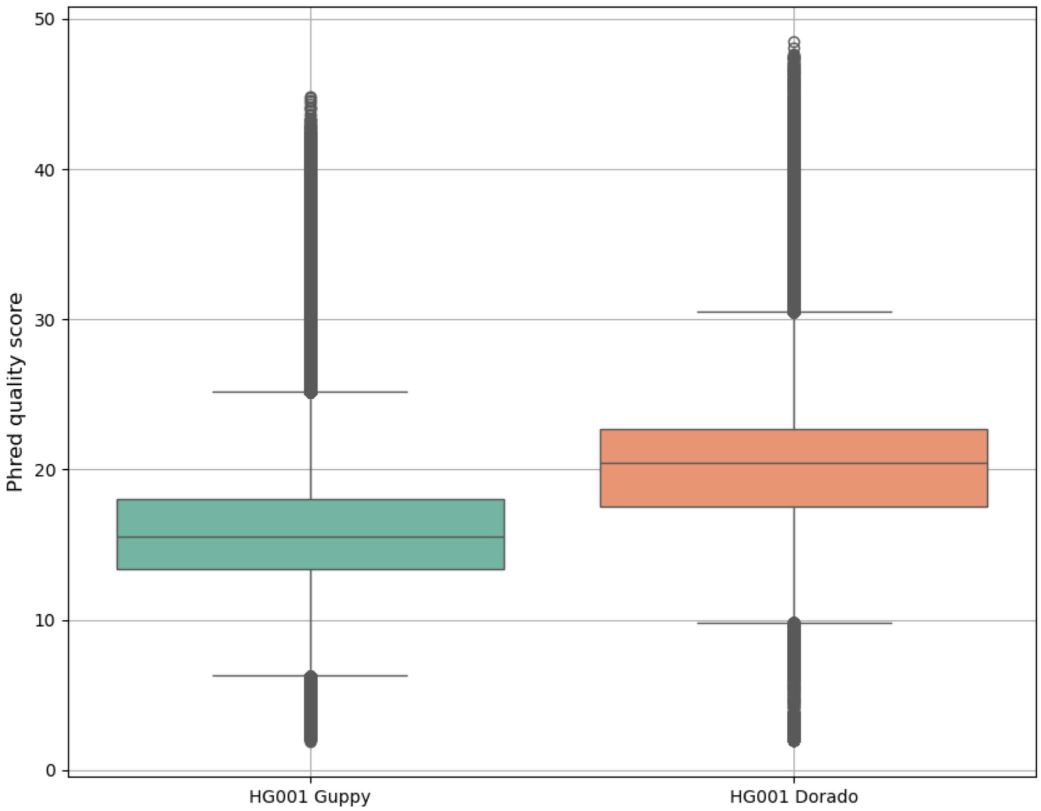

Figure S.1 Illustration of the improvement in the raw read quality scores obtained by repeating basecalling of the raw squiggle data using Dorado (v0.9.0.). Guppy basecalling was performed using version 6.5.7-gpu.

1.2.2 Supplementary Figure S2

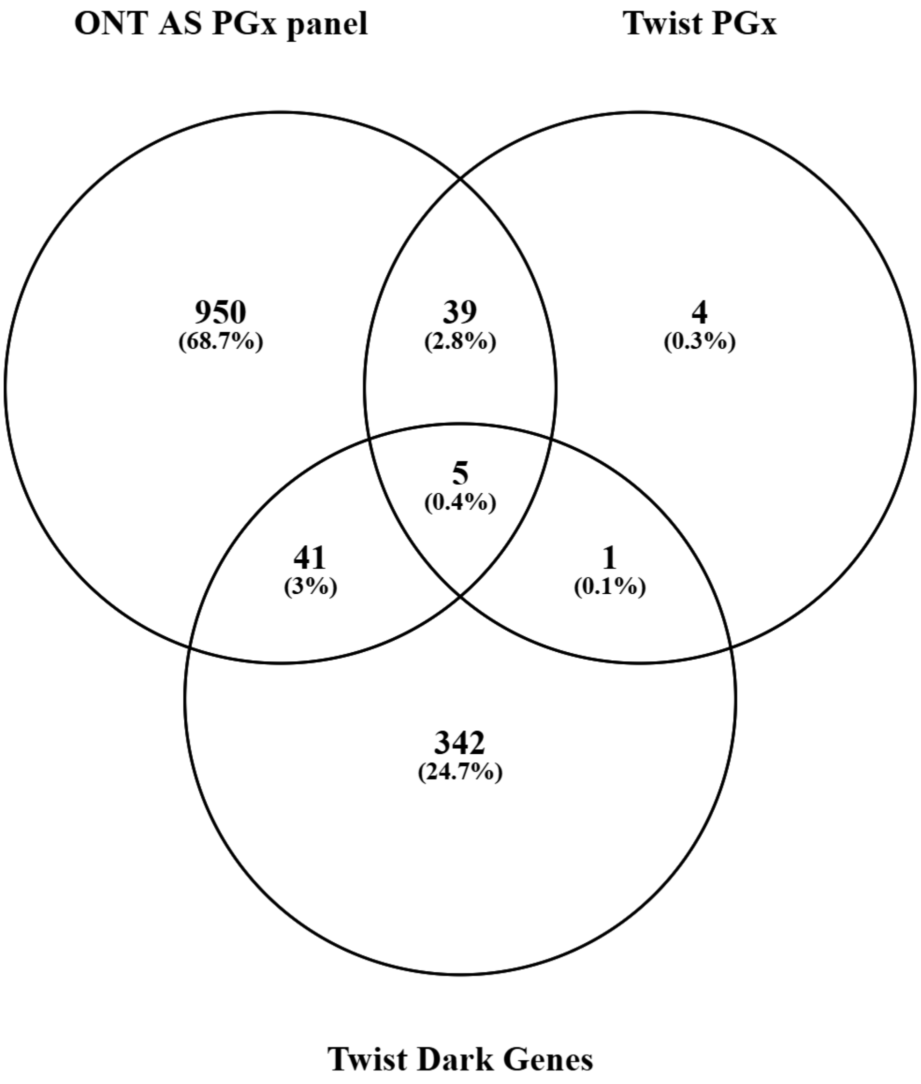

Figure S.2 Venn-diagram illustrating the overlap in the number of genes assayed in the ONT AS PGx panel, the Twist Alliance PGx panel, and the Twist Alliance Dark Genes panel. Diagram crated using Venny (Oliveros, J.C. (2007-2015) Venny. An interactive tool for comparing lists with Venn's diagrams. https://bioinfogp.cnb.csic.es/tools/venny/index.html).

1.2.3 Supplementary Figure S3

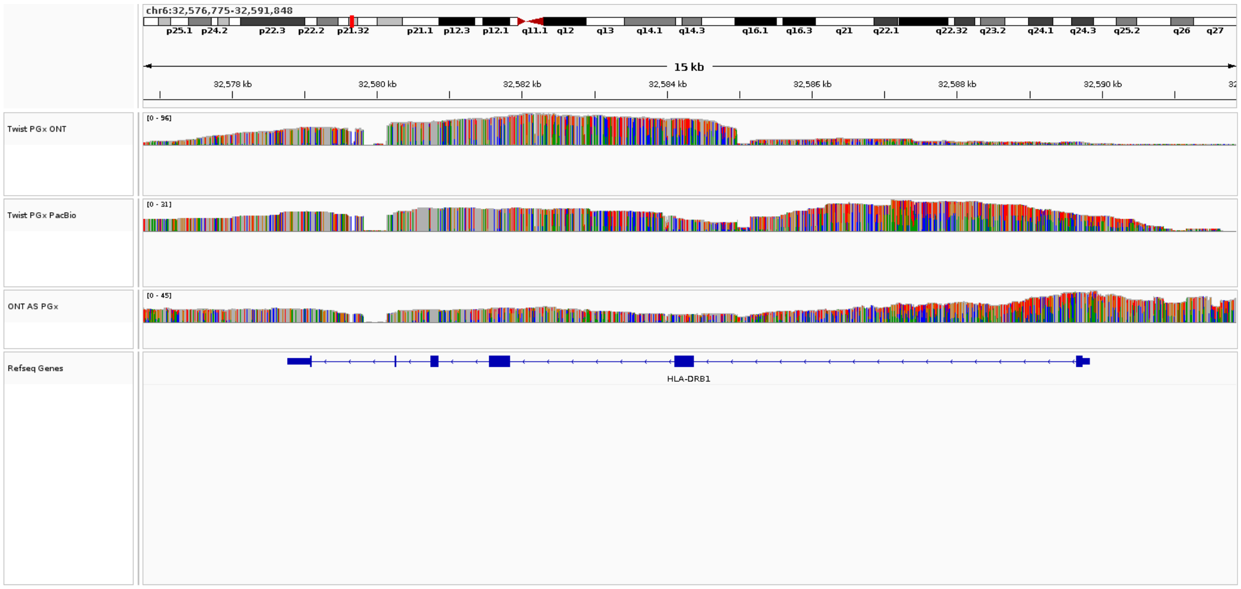

Figure S.3 IGV representation of the sequencing depth along the *HLA-DRB1* gene obtained through the Twist Alliance long-read PGx panel, as sequenced on ONT (top) and PacBio Sequell II (middle) compared to our ONT AS PGx panel (bottom). The drop in sequencing depth at the beginning of the gene in the top Twist PGx ONT dataset might explain the 'No Reads' call obtained during diplotyping with StarPhase.

1.2.4 Supplementary Figure S4

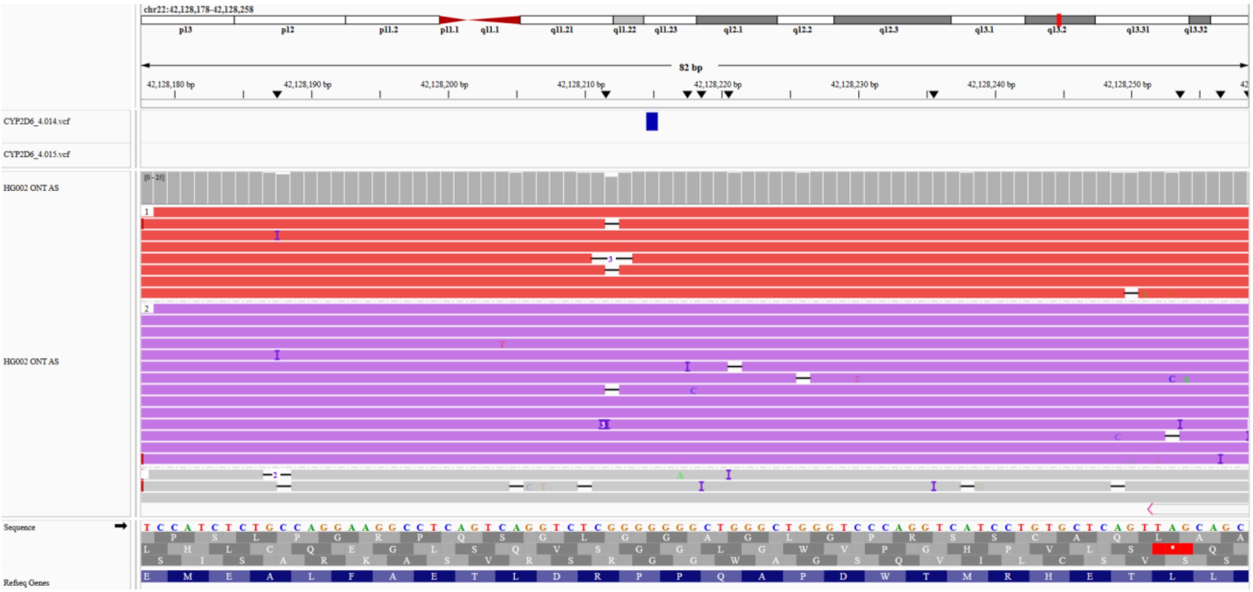

Figure S.4 IGV screenshot illustrating the rs1473203326 variant in CYP2D6*4.014 minor allele (top, blue rectangle) and its absence in CYP2D6*4.015 minor allele (top). Despite the StarPhase 4*0.14 call in sample HG002, this variant could not be confirmed by manually querying the aligned reads (middle, red: allele 1, purple: allele 2). This variant is located within a guanosine homopolymer stretch, which might be impacting this result.

1.2.5 Supplementary Figure S5

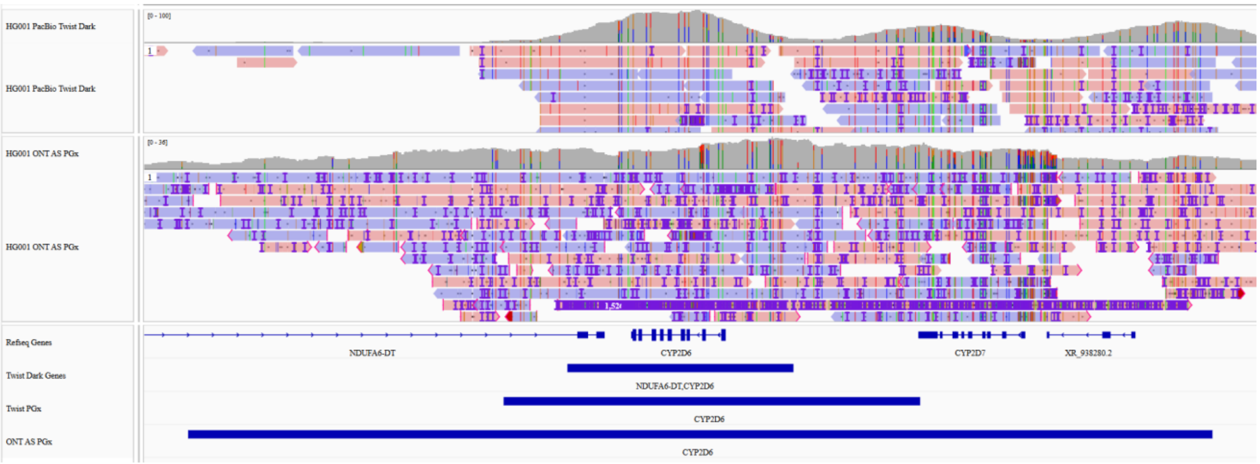

Figure S.5 IGV screenshot illustrating the CYP2D6 gene locus along with the read alignments of the HG001 reference sample, as sequenced on PacBio Revio using the Twist Alliance Dark Genes panel (top) and the ONT PromethION using our ONT AS PGx panel (middle). In the bottom panel, the capture regions of the Twist Dark Genes, Twist PGx, and our ONT AS PGx panel for CYP2D6 are shown (blue bars). Due to the limited capture region in the Twist Dark Genes panel, the output StarPhase CYP2D6 call based on these alignments is false, while it is correct in the ONT AS PGx setup.

1.2.6 Supplementary Figure S6

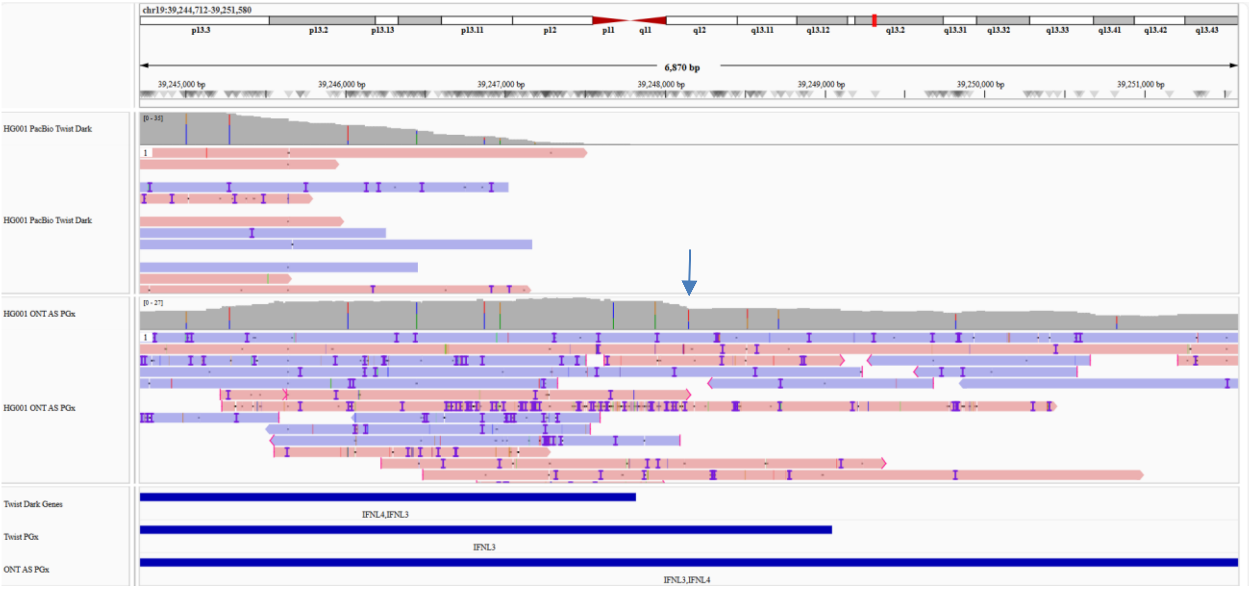

Figure S.6 IGV screenshot illustrating the wrong IFNL3 call based on the Twist Alliance Dark Genes panel for reference sample HG001 (top). The rs12979860 is indicated by the arrow. Using the ONT AS PGx panel (middle), the star-allele call is called correctly. Due to the lack of reads covering this variant locus in the Twist Dark Genes panel, the star-allele call is incorrect. The bottom part of the figure illustrates the captured region for each of the panels (horizontal blue bars).

1.2.7 Supplementary Figure S7

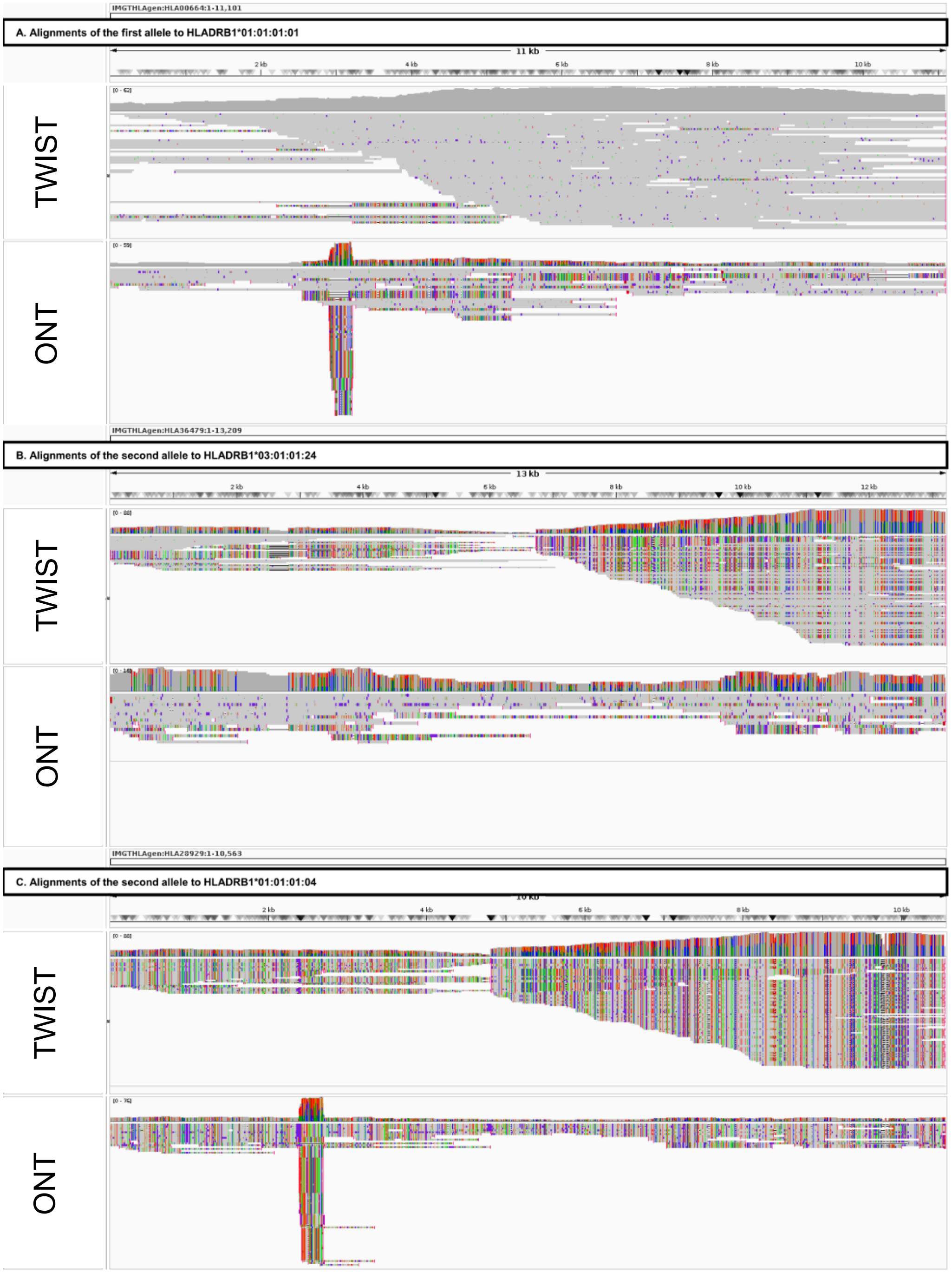

Figure S.7 Manual curation of the *HLA-DRB1* gene for the HG001 sample. Both for the Twist Dark Genes and ONT AS PGx strategy, the alignments to the *HLA-DRB1* gene were extracted from the respective .bam files and re-aligned in a haplotype-specific way to the HLA-DRB1*01:01:01:01 (top), HLA-DRB1*03:01:01:24 (middle), and HLA-DRB1*01:01:01:04 (bottom) alleles using minimap2. Their reference sequences were obtained from the IPD-IMGT/HLA database. The presence of three reads spanning almost the complete HLA-DRB1*03:01:01:24 allele in the ONT AS PGx dataset might have resulted in its correct call, where the Dark Genes panel identified the wrong allele.

1.2.8 Supplementary Figure S8

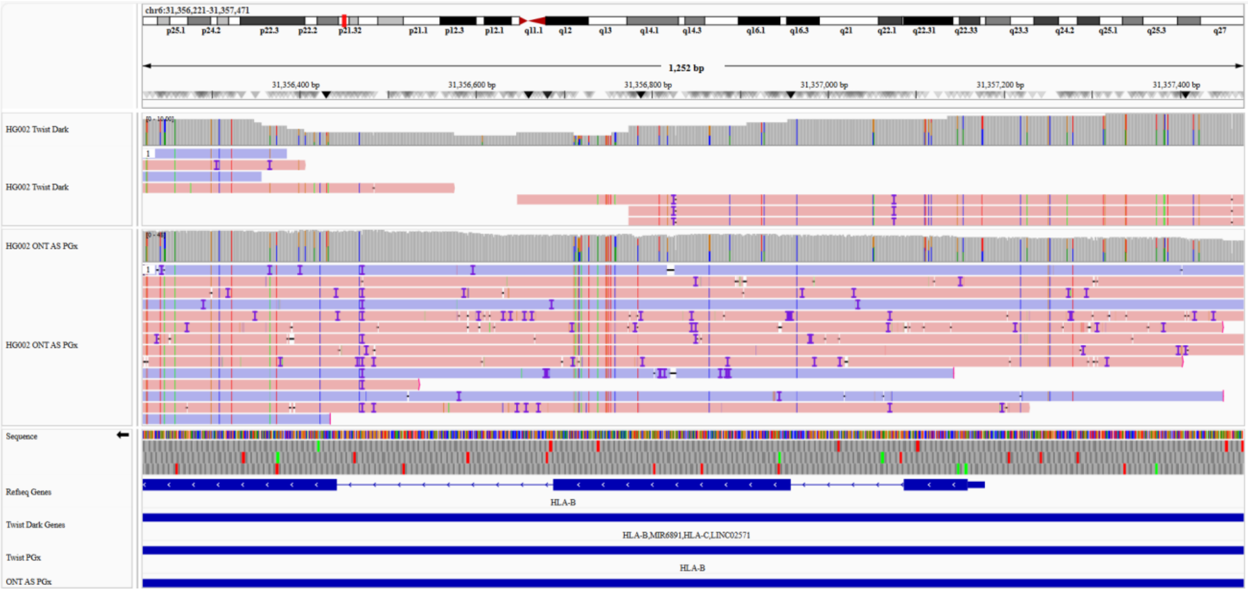

Figure S.8 IGV screenshot illustrating the HG002 alignments for the Twist Alliance Dark Genes panel (top), and the ONT AS PGx panel (middle), zoomed in on the first exons of HLA-B. The capture regions for the Twist Dark Genes, Twist PGx, and ONT AS PGx panel are shown in the bottom (blue bars). Due to limited sequencing depth for HLA-B exon 2, StarPhase most probably calls the wrong HLA-B allele in the PacBio Dark Genes data.

1.3 Supplementary Notes

1.3.1 Supplementary Note S1

While we did not include the MT-RNR1 gene in the ONT AS PGx panel, the MT-RNR1 gene has some sequencing depth in our sequencing data. Apart from some very short reads that remained present in our ONT AS dataset, we retrieved reads in our data of which the first part aligned to a gene of interest, and the clipped-end sequence supplementary aligned to the mitochondrial genome (cf. Figure below). As the AS algorithm recognizes the first 400-800 bases of the read before a rejection decision is made, these initial bases can overlap with a specified gene-of-interest and thus be accepted for further sequencing. We therefore believe that these reads are genuine nuclear DNA fragments rather than library preparation artifacts. Therefore, caution should be taken in genotyping mitochondrial DNA variants from whole genome sequencing data. During evolution, mitochondrial DNA like sequences have been transferred into the nuclear genome (NuMTs). Tao et al. (2023) have identified 958 NuMTs in the nuclear T2T-CHR13 human reference genome, spanning up to 90% of the entire mitochondrial genome. This could result into the alignment of fragmented nuclear NuMT DNA to the mitochondrial genome and vice versa. Therefore, as there is no guarantee on the mitochondrial origin of these aligned reads, we did exclude the *MT-RNR1* gene from the ONT AS PGx results in the comparison discussed below. In contrast, as the *MT-RNR1* capture probes were spiked in the Twist PGx dataset for this benchmarking dataset, and achieved significantly high sequencing depth in line of what should be expected given its higher copy number, it could be assumed that most of the reads in this dataset do origin from the mitochondrial genome, although coincidental co-capturing of NuMT cannot be excluded.

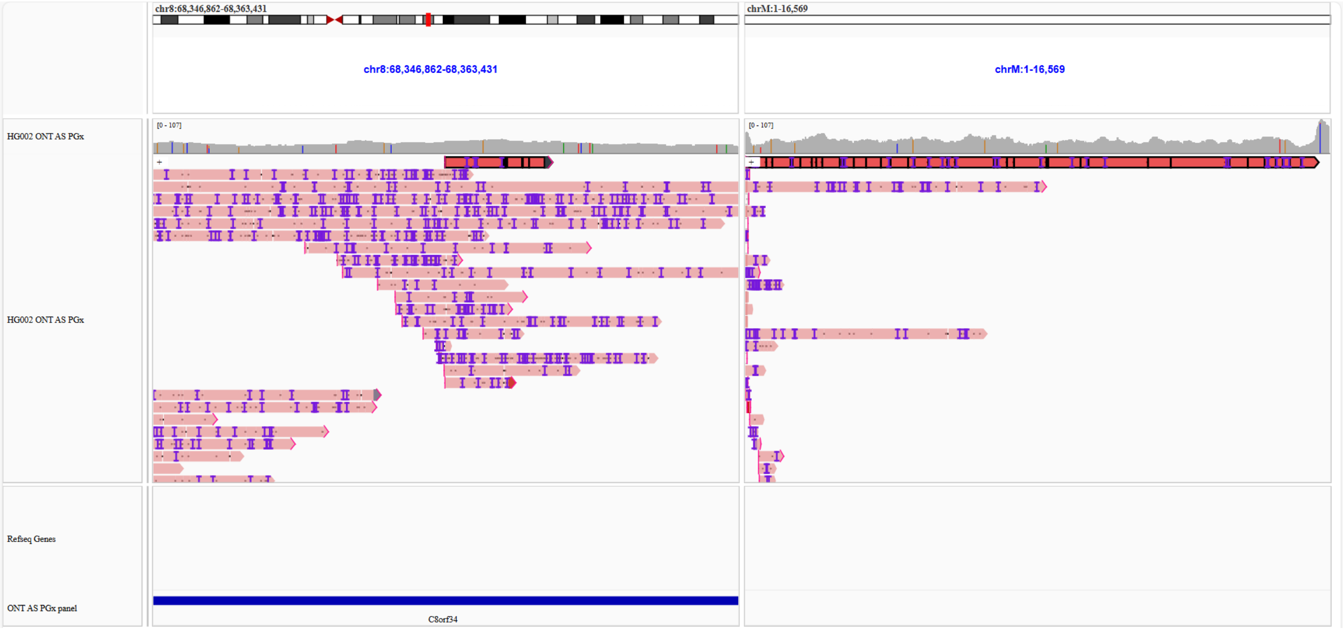

Figure IGV screenshot illustrating the almost complete mapping of a read to the mitochondrial genome chrM (right panel), despite it not being part of the ONT AS PGx panel. As illustrated in the left panel, the first part of that read aligned to *C8orf34*, which is part of the ONT AS PGx panel. This finding illustrates the integrating of parts of the mitochondrial genome within the nuclear genome.
